# Hemophagocytic Lymphohistiocytosis With Obstructive Jaundice as a Rare Presentation of Disseminated Tuberculosis in an Adult

**DOI:** 10.7759/cureus.38875

**Published:** 2023-05-11

**Authors:** Kotha Vamshikrishnapatel, Ratnadeep Biswas, Vishnu S Ojha, Aniketh V Hegde, Vijay Kumar

**Affiliations:** 1 Internal Medicine, All India Institute of Medical Sciences, Patna, IND

**Keywords:** anti-tubercular agents, dysregulated immune disorder, hemophagocytic lymphohistiocytosis, disseminated tuberculosis, tuberculosis, pancytopenia, bone marrow, immunity, obstructive jaundice

## Abstract

Tuberculosis (TB) is a disease of global concern due to its varying clinical presentations and outcomes. Hemophagocytic lymphohistiocytosis (HLH) syndrome, along with obstructive jaundice, is one of the rarest presentations of tuberculosis involving immune activation and has a very high mortality rate. Thus, on-time diagnosis becomes crucial for the management of the disease. Prompt treatment with anti-tubercular therapy (ATT) can limit the morbidity and mortality associated with it.

We report the case of a 28-year-old male who presented with fever, yellowish discoloration of the skin, features of bicytopenia, jaundice with hepatosplenomegaly, and ascites. The liver function test (LFT) was suggestive of obstructive jaundice. TB was confirmed on the analysis of lymph node aspirates, and the contrast-enhanced computed tomography (CECT) of the thorax and abdomen was suggestive of disseminated tuberculosis. Upon investigation, the criteria for HLH were fulfilled. Bone marrow aspiration smears revealed multiple hemophagocytic histiocytes in the background of a hypercellular marrow, erythroid hyperplasia, and myeloid-to-erythroid ratio of 1:1. Thus, a diagnosis of disseminated TB with HLH and obstructive jaundice was established. A modified ATT regimen was started, keeping in mind the deranged LFT of the patient, but no immunosuppressive therapy was initiated as it could make the TB worse. This case demonstrates the fact that in cases of hemophagocytic syndrome with tuberculosis as an underlying cause, just starting ATT without immunosuppression could be rewarding and lifesaving.

## Introduction

Tuberculosis (TB) is a disease of global concern due to its varying clinical presentations and outcomes. From 2018 to 2022, approximately 19.8 million people were treated for TB [1]. Out of the 15% of all tuberculosis cases, which are extrapulmonary tuberculosis, 20% end up with disseminated tuberculosis [2,3]. Disseminated TB is a life-threatening complication of tuberculosis with the hematogenous dissemination of *Mycobacterium tuberculosis* (MTB) to various organs of the body. It manifests with a plethora of clinical symptoms ranging from chronic constitutional symptoms, jaundice, seizures, and ascites to even multiorgan failure [2]. Thus, on-time diagnosis becomes crucial for the management of the disease. The diagnosis of disseminated TB is made based on a positive polymerase chain reaction, the bacteriological isolation of MTB, or the finding of a caseating granuloma on histology from bone marrow, the liver, blood, or any two noncontiguous organs supported by a radiological finding.

Addressing a widespread infection by MTB poses a great challenge to the immune system, which involves the activation of multiple pathways and various cell types in an attempt to contain the infection [4]. Such conditions of incredible stress on the immune system pave the way for the dysregulation or abnormal activation of some immunological pathways, which can cause a host of complications.

One such life-threatening complication is hemophagocytic lymphohistiocytosis (HLH). It is a potentially fatal hyperinflammatory disorder involving excessive immune activation resulting in hemophagocytosis, inflammation, and tissue damage [5]. It presents as a multisystemic inflammatory syndrome caused due to excessive cytokine release by activated cytotoxic T cells and natural killer cells [6]. It can be broadly classified as either primary (inherited) or secondary (acquired). Primary HLH frequently manifests in infancy and results from a hereditary susceptibility [7]. Secondary HLH can occur in association with infections, most commonly viral infections, but can also be associated with fungal, parasitic, and bacterial infections, including TB [8]. At first, the symptoms may resemble typical infectious diseases such as fever of unknown origin and hepatitis, with findings such as swollen lymph nodes and an enlarged spleen. However, as the condition progresses, it could lead to dangerous drops in white blood cell counts that can be life-threatening. The mortality of HLH can be as high as 50%, a figure that is believed to only increase with delays in diagnosis [9].

The current treatment guidelines for primary HLH mainly comprise chemo-immunotherapeutic drugs. However, in HLH cases secondary to infections, treating the infection may be equally or even more effective by removing the inciting factor behind the immune dysregulation [10]. Thus, proper treatment can significantly reduce morbidity and mortality. We report this case to highlight the fact that the secondary causes of HLH in adults, particularly tuberculosis, can be successfully treated solely with the use of anti-tubercular therapy (ATT).

## Case presentation

A 28-year-old male from Bihar, a state in eastern India, presented to the general medicine outpatient department with complaints of fever and cough for three months, associated with a history of yellowish discoloration of the skin and edema in the lower limbs for 14 days. The patient experienced high-grade, continuous fevers associated with chills, dry cough, gradually increasing jaundice, clay-colored stools, loss of appetite, and significant weight loss (10 kg in three months). He had been transfused with three units of packed red blood cells in view of anemia two months ago. He did not have any other significant past history or medication history. He had not traveled anywhere recently. He had received the birth dose of the bacille Calmette-Guérin (BCG) vaccine. The patient had no history of alcohol intake. On examination, the patient was poorly nourished and feverish with tachycardia. Pallor, icterus, and bilateral pedal edema were present, whereas clubbing and cyanosis were absent. Firm, non-tender lymph nodes measuring around 1.5 cm were palpable in the left posterior triangle of the neck, whereas axillary, cervical, and inguinal lymph nodes were not palpable. Petechial rashes were noticed on the lower extremities. The neck veins were engorged, but the jugular venous pressure was not raised. The gastrointestinal examination revealed abdominal distension and mild hepatosplenomegaly. Shifting dullness was also elicited. The examination of other systems was unremarkable.

At the time of presentation, the patient's blood pressure was 115/67 mmHg, and oxygen saturation was 94% in room air. The hematological evaluation showed anemia (a red blood cell count of 3.2 million cells per microliter and a hemoglobin level of 8.5 g/dL), thrombocytopenia (a platelet count of 70,000 cells per microliter), a normal total leucocyte count (6,700 cells per microliter), lymphopenia, raised C-reactive protein, hypertriglyceridemia (450 mg/dL), and hyperferritinemia (1,650 mg/dL). Liver function tests (LFT) were deranged, which showed a higher level of total bilirubin (13 mg/dL), with direct and indirect fractions of 7.9 mg/dL and 5.2 mg/dL, respectively. There was a mild elevation in aspartate aminotransferase (60 U/L) and alanine transaminase (58 U/L), whereas a significant rise in alkaline phosphatase (ALP) (1,800 U/L) was noted, suggestive of cholestasis. Lymph node aspiration was done from the cervical nodes, and on a cartridge-based nucleic acid amplification test (Xpert MTB/RIF assay, Cepheid Inc., Sunnyvale, CA), a rifampicin-sensitive *Mycobacterium tuberculosis* complex was identified. The rapid kit tests for hepatitis B surface antigen and antibodies to hepatitis C virus were nonreactive.

An ultrasound of the abdomen revealed intrahepatic biliary radicle dilatation (IHBRD). Contrast-enhanced computed tomography (CECT) of the abdomen and thorax visualized multiple enlarged necrotic nodes in the cervical, mediastinal, axillary, and retroperitoneal regions with multiple centrilobular nodules, ground glass opacities, consolidation, and a few cavitary lesions seen in bilateral lung parenchyma suggestive of disseminated TB. A mild bilateral pleural effusion, moderate ascites, and hepatosplenomegaly were also present. Gallstones were not detected, but dilated intrahepatic biliary radicles were visualized. In the porta hepatis area, an enlarged lymph node measuring approximately 2 cm was observed. This enlargement was attributed to the disseminated TB infection, and it was believed to be responsible for the obstruction of the biliary system.

**Figure 1:**
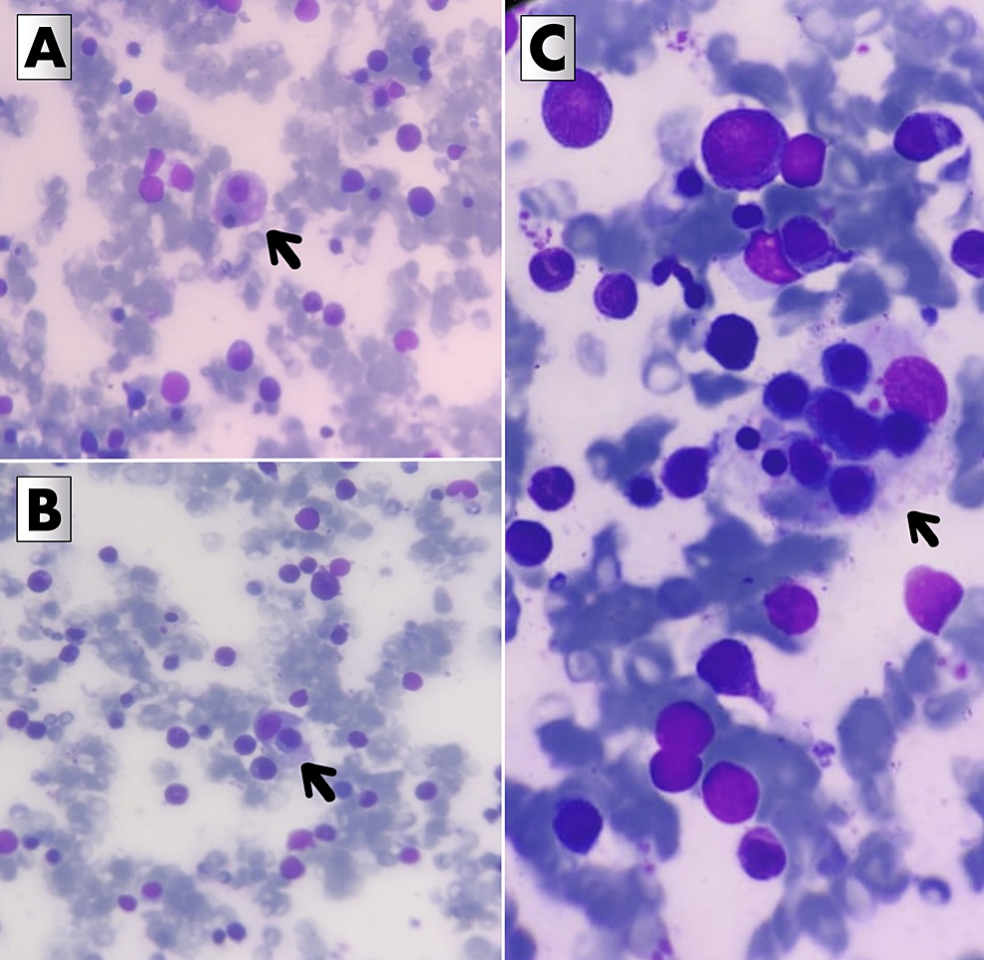
Bone marrow aspirate smears showing hemophagocytic histiocytes (Giemsa stain) (A) The arrow points toward a histiocyte that has phagocytosed an erythroid cell. (B) The arrow points toward a histiocyte that has phagocytosed a lymphoid cell. (C) The arrow points toward a histiocyte that has phagocytosed multiple cells from different lineages

Bone marrow aspirate smears revealed hypercellular marrow with erythroid hyperplasia and a myeloid-to-erythroid ratio of 1:1. No increase in blasts was noted. Abundant hemophagocytic histiocytes were appreciated, as shown in Figure 1.

The diagnosis of disseminated TB with HLH and obstructive jaundice was established based on clinical, histological, microbiological, and radiological impressions. The prescription of immunosuppressant medications for HLH was strongly deferred as it may flare up the tuberculosis infection, which is the main trigger for HLH. Therefore, a modified ATT regimen (consisting of levofloxacin 750 mg once daily, ethambutol 900 mg once daily, and linezolid 600 mg once daily) was started due to the deranged LFT.

During the hospital stay and after the initiation of treatment, the patient's LFT improved. Once the bilirubin was under 5 mg/dL, isoniazid and rifampicin were started at half the usual dose and gradually titrated toward the normal dose based on serial LFT monitoring. The patient was not subjected to further investigations, such as magnetic resonance cholangiopancreatography (MRCP), to determine the cause of the obstructive jaundice, since the ALP levels decreased drastically after the ATT. Ursodeoxycholic acid, vitamin E, and vitamin K doses were given during the course of his stay. When serum bilirubin and alkaline phosphatase levels were below 2 mg/dL and 600 U/L, respectively, pyrazinamide was also added to the treatment regime. After completing three months of ATT, the patient's ALP levels decreased to 120 U/L, demonstrating significant improvement. Additionally, the patient's pancytopenia had resolved. ATT was continued for a duration of one year.

## Discussion

Disseminated tuberculosis is a condition involving the lymphohematogenous spread of MTB to various extrapulmonary sites [11]. Thus, it involves the recruitment of various immune cells. This can lead to the secondary activation of various diseases. One of those acquired diseases is HLH. It is a disorder of the immune system that is characterized by abnormal and life-threatening activation of primarily cytotoxic T-lymphocytes. This condition results in the release of excessive cytokines and triggers the activation of macrophages [12]. It is characterized by fevers, lymphadenopathy, splenomegaly, cytopenias, hyperferritinemia, hypertriglyceridemia, hypofibrinogenemia, low or absent natural killer (NK) cell activity, and high-soluble cluster of differentiation 25 (CD25) (interleukin 2 receptor alpha) levels, along with a demonstration of hemophagocytosis. The underlying mechanism of tuberculosis with hemophagocytic lymphohistiocytosis (TB-HLH) remains unclear, although it is generally believed that TB infection causes an imbalance in the immune system's stability, which can result in the development of secondary HLH [13]. Inflammatory mediators such as interferon gamma (IFN-γ), tumor necrosis factor-alpha (TNF-α), granulocyte-monocyte colony-stimulating factor (GM-CSF), and interleukins 1, 6, and 18 are crucial indicators in HLH patients with cytokine storm. Following phagocytosis, MTB is thought to induce type 1 T helper (TH1)-mediated cytotoxicity along with the activation of natural killer cells and macrophages, which may result in a cytokine storm, giving rise to HLH. Furthermore, it has been postulated that MTB may lead to a consistent cytokine release by activated macrophages in lymph nodes via an interleukin 12- and interleukin 15-mediated mechanism [14].

Since there is no definitive test for HLH, it is often diagnosed clinically when five out of the eight proposed criteria by the International Histiocyte Society are fulfilled: (1) fever, (2) enlarged spleen, (3) bicytopenia (decreased cell counts in two cell lines) (with hemoglobin levels less than 9 g/dL, absolute neutrophil count less than 1,000 cells per microliter, and platelet count less than 100,000 cells per microliter), (4) hypertriglyceridemia (more than 265 mg/dL) and/or hypofibrinogenemia (less than 150 mg/dL), (5) hyperferritinemia (more than 500 μg/L), (6) finding of hemophagocytosis, (7) decreased or no activity of the natural killer cells, and (8) soluble CD25 levels more than 2,400 units per milliliter [15]. In our case, six out of the eight criteria were satisfied, pointing toward the diagnosis of HLH. Hemophagocytosis was demonstrated on aspiration samples from bone marrow. Elevated levels of serum bilirubin and ALP, combined with the clinical manifestation of jaundice and the dilatation of intrahepatic biliary radicles, indicated the presence of obstructive jaundice. The underlying pathophysiology behind obstructive jaundice secondary to tuberculosis may be due to the inflammation and strictures of the main biliary duct, the compression of the bile duct, a tubercular mass on the pancreatic head, or a tuberculous abscess in the retroperitoneum [16]. In this patient, it is highly likely that the enlarged lymph node near the liver hilum was causing the compression of the biliary system, leading to the development of obstructive jaundice.

Since HLH and disseminated TB are potentially fatal diseases, a delay in treatment may be life-threatening. According to Schippers et al., if patients with TB-HLH do not receive ATT, all of them die; however, combining ATT with immunotherapy can lower the mortality rate by 40%-60% [17]. Some academics hold the belief that promptly commencing and efficiently administering ATT are crucial in averting HLH among individuals with TB and are the fundamental aspect of managing TB-HLH [13]. According to a literature review conducted by Brastianos et al. on 36 similar cases, it was observed that patients who received only anti-TB therapy had better survival rates than those who were administered a mix of anti-TB drugs and different immunomodulatory therapies [9]. Similarly, in our case, ATT was the mainstay of the treatment without any immunosuppressive drugs. The initiation of ATT was particularly challenging in this case due to the deranged LFT resulting from obstructive jaundice. Thus, levofloxacin, ethambutol, and linezolid were started, which led to a dramatic improvement in the patient. After the stabilization of the liver functions, isoniazid, rifampicin, and eventually pyrazinamide were also added serially, leading to a progressive resolution of symptoms.

Thus, the possibility of tuberculosis being the inciting etiology should be considered in patients having secondary HLH, and therefore, treatment with the specific agent is recommended. The early identification of triggers may prevent the use of immunosuppressive therapy alone, which may entail a risk of a flare-up reaction due to an underlying etiology, such as TB in this case.

## Conclusions

If patients exhibit symptoms such as fever, jaundice, hepatosplenomegaly, enlarged lymph nodes, cytopenia, hyperferritinemia, and hypertriglyceridemia, clinicians should consider the possibility of HLH. In such cases, investigations to confirm the diagnosis and determine the underlying cause should be conducted, which can assist in deciding on appropriate treatment strategies. HLH is a rare complication that can occur in cases of disseminated TB. If HLH is suspected to be associated with TB, it is important to avoid using only immunosuppressive agents for therapy as it could make the TB worse. Instead, treatment with ATT should be aggressively initiated, as early treatment is crucial in reducing illness and death, particularly in countries where TB is common.
